# Inhibition of influenza A virus infection by ginsenosides

**DOI:** 10.1371/journal.pone.0171936

**Published:** 2017-02-10

**Authors:** Wei Dong, Amber Farooqui, Alberto J. Leon, David J. Kelvin

**Affiliations:** 1 Division of Immunology, International Institute of Infection and Immunity, University Health Network & Shantou University Medical College, Shantou, China; 2 Division of Experimental Therapeutics, Toronto General Hospital Research Institute, University Health Network, Toronto, Ontario, Canada; 3 Guangdong Provincial Key Laboratory of Infectious Diseases and Molecular Immunopathology, Shantou, China; 4 Institute of Medical Science, Faculty of Medicine, University of Toronto, Toronto, Ontario, Canada; 5 Deptartment of Immunology, Faculty of Medicine, University of Toronto, Toronto, Ontario, Canada; China Academy of Chinese Medical Sciences, CHINA

## Abstract

Influenza viruses cause mild to severe respiratory infections in humans. Due to efficient means of transmission, the viruses infect human population on a large scale. Apart from vaccines, antiviral drugs are used to control infection; neuraminidase inhibitors are thought to be the first choice of treatment, particularly for severe cases. Rapidly evolving and emerging influenza viruses with increased frequency of viral resistance to these drugs stress the need to explore novel antiviral compounds. In this study, we investigated antiviral activity of ginseng extract and ginsenosides, the ginseng-derived triterpene and saponin compounds, against 2009 pandemic H1N1 virus in vitro and in vivo. Our data showed that treatment of mice with ginsenosides protected the animals from lethal 2009 pandemic H1N1 infection and lowered viral titers in animal lungs. Mechanistic studies revealed that ginsenosides interact with viral hemagglutinin protein and prevent the attachment of virus with α 2–3’ sialic acid receptors present on host cell surfaces. The interference in the viral attachment process subsequently minimizes viral entry into the cells and decreases the severity of the viral infection. We also describe that sugar moieties present in ginsenosides are indispensible for their attachment with viral HA protein. On the basis of our observations, we can say that ginsenosides are promising candidates for the development of antiviral drugs for influenza viruses.

## Introduction

Influenza A viruses primarily cause respiratory tract infections in mammals and birds. The H1N1 and H3N2 influenza virus subtypes are the most prevalent in humans and account for several pandemics, including the 2009 H1N1 pandemic that caused worldwide mortality and morbidity in the recent past [1]. Additionally, avian and other influenza viruses such as H7N7, H5N1, [2] H7N9 [3] and H10N8 [4] are also reported to cause fatal disease in humans. There are two major types of antiviral drugs currently in clinical use: neuraminidase inhibitors (NAIs) such as oseltamivir, peramivir, zanamivir, laninamivir and the M2 ion-channel blocker (amantadine, rimantadine). The latter, however, are not widely used for treatment because of widespread resistance across the species. It is evident that progress in the field of antiviral drug discovery lags behind today’s challenges since only one antiviral agent, DAS181, a novel sialidase protein is in phase II clinical trials [5]. The situation demands antiviral testing of novel compounds in rapid fashion.

The herb ginseng (*Panax quinquefolium*) is widely used as a symptom reliever for respiratory viral infections such as influenza [6]. The plant contains a pharmacologically active series of triterpenes and saponins called ginsenosides which are composed of a four trans-ring rigid steroid skeleton attached with various sugar motifs [6]. According to the position of the sugar residues, they can be classified into two major groups, 20(s) protopanaxadiol (PPD) and 20(s) protopanaxatriol (PPT), that have shown antineoplastic and antioxidant properties on a variety of cells [7–9].

It has been reported that ginsenosides have the potential to against coxsackievirus B3, enterovirus 71, human rhinovirus 3 and haemagglutinating virus of Japan (HVJ) infection (1)(2), however, the antiviral effect of ginsenosides on influenza and the mechanism involved in the protection have not yet been explored.

In this study, we discovered the novel anti-influenza properties of ginseng and ginseng-derived compounds in vitro and in vivo. We observed that ginsenoside compounds, particularly Rb1, interact with influenza A virus surface hemagglutinin (HA) and interfere in viral binding with cell surface receptors, thus inhibiting viral entry in host cells.

## Materials and methods

### Cells, virus and antiviral compounds

Madin-Darby canine kidney (MDCK) and Chinese hamster ovary (CHO) cells, obtained from ATCC, USA were cultured in Dulbecco’s Modified Eagle Medium (DMEM, M&C Gene Technology) supplemented with 10% fetal bovine serum, 10 mM L-glutamine, 100U penicillin/ml and 100μg/ml streptomycin, at 37°C in 5%CO_2_. A/Nanchang/8002/2009 H1N1 (NC2), A/Puerto Rico/8/34 H1N1 (PR8) and A/Hong Kong/1/1968 H3N2 (HK68) virus strains were used. NC2 was an A/California/07/2009-like 2009 pandemic H1N1 (2009pdm H1N1) virus that we isolated from a nasopharyngeal (NP) swab of a patient admitted to a local hospital in Nanchang, Jiangxi province of China, in December 2009 [10]. Ginseng extract (GE) was prepared by dissolving a capsule of ginseng, purchased from local pharmacy, in sterile MilliQ water. Ginsenosides Rb1, Rb2, Rb3, Rg1, Rg3, PPD, PPT, Rh2, g-PPD and g-PPT were purchased from Fleton Co. (Chengdu, China) and dissolved in sterile MilliQ water to prepare stock solutions with a final concentration of 10mg/ml and stored at -20°C.

### Animals

Eight- to ten-week-old female BALB/c mice were purchased from Vital River Laboratories, Beijing, China and maintained on standard feed and water to acclimate in a specific pathogen-free (SPF) facility. For the infection study, animals were kept in micro-isolator cages under ABSL2 conditions. All procedures were performed in a certified Class II biosafety cabinet according to the guidelines approved by the institutional Animal Care and Use Committee. The ethical committee of Shantou University Medical College, China, approved the study.

### In vitro antiviral assays

In a post-treatment assay, cells were infected with NC2 at multiplicity of infection (MOI) 1 followed by the addition of viral DMEM (vDMEM) containing 2μg/ml TPCK (L-1-tosylamide-2-phenylethyl chloromethyl ketone) trypsin (Sigma) and GE.

In a virus-pretreatment assay, NC2 was pre-incubated with GE for 1 hour at 37°C and subsequently used for infection. In a cell-pretreatment assay, MDCK cells were cultured in vDMEM and GE 2 hours prior to infection. The monolayer was infected with NC2 at MOI 1 and cell supernatants were collected after 24 hours of incubation and titrated for viral loads using the TCID_50_ assay [11].

In the viral attachment assay, 2009pdm H1N1 virus was pretreated with 500 μg of Rb1 or vDMEM for 1 hour at 37°C. CHO cells were then infected with this mixture for 1 hour at 4°C that only allows virus adsorption to the cell surface. RNA was extracted from cells using Trizol and real time RT-PCR targeting the viral M gene was performed. In the replication assay, CHO cells were infected with Rb1 or vDMEM-pretreated 2009pdm H1N1 virus at 37°C. Cells were collected 10 and 24 hours post infection and subjected to real time RT-PCR [10].

### Hemagglutination assay

The hemagglutination assay was performed to evaluate the effect of the GE and ginsenosides on virus adsorption to target cells. Compounds were mixed with an equal volume of serially diluted influenza virus. After 1 hour of incubation at 37°C, 50μl of the mixture was added to 0.5% chicken red blood cells (CRBCs) and incubated for 30 minutes at room temperature. The lowest amount of viral particles able to agglutinate with CRBCs in the presence of ginsenosides was compared with a mock control containing no antiviral compound.

### Animal experiments

For animal experiments, 10^3^ EID_50_ of viral inoculum was pre-incubated with equal volumes of GE, Rb1 or other ginsenosides (2mg/ml) at 37°C for 1 hour. The virus and compound mixture was administered to naïve animals intranasally under anesthesia, which was induced by the administration of 400mg/kg of 2, 2, 2-tribromoethanol (Sigma). For dose dependent assays, different concentrations of Rb1 were used in the same manner. Compound-virus mixtures were prepared with 10^5^ EID_50_ of NC2 to examine antiviral activity of Rb1 at a high viral dose. In the case of mock controls, the virus-vehicle mixture was prepared in HBSS in a similar manner. In one group, 80mg/kg of GE was given by oral gavages twice daily from day 0 to day 7 post-infection and animals were infected with 10^3^ EID_50_ of NC2. Weight loss, mortality and morbidity of animals were recorded every day up to 15 days. Animals were sacrificed upon a body weight loss of 20%. Animal euthanization was performed by excessive exposure to carbon dioxide gas. For the determination of viral loads, lung tissues were collected on days 3 and 6 post infection (p.i.) from those animals who did not reach to the humane endpoint of 20% loss in body weight [11].

### Immunoblot assay

Different concentrations of ginsenosides Rb1 were mixed with recombinant HA protein -Influenza A virus H1N1 (A/California/07/2009) (Sino Biological Inc., Beijing, China) and incubated at 37°C for 1 hour and then was spotted onto PVDF membrane. Immunoblotting was performed with Influenza A virus HA antibody (Santa cruz) and secondary goat anti-mouse IgG peroxidase conjugate (Calbiochem) and visualized by enhanced chemiluminescence (ECL).

### ELISA

ELISA was performed with recombinant HA proteins of the influenza viruses such as H1N1 and H7N9 which are known for their binding specificities to α2–6 and α2–3 sialic acid receptors respectively. Influenza A Hemagglutinin/HA ELISA Pair Set kits (Sino Biological, China) were used to determine the binding of Rb1 with HA proteins. The experiment was performed as the protocol described by the manufacture. Briefly, HA capture antibody was coated onto a 96-well plate followed by the addition of 200pg/ml of purified HA protein pre-absorbed with Rb1 or HBSS for 1 hour. HRP-labeled anti-H1N1 and H7N9 polyclonal antibody was used for detection per the protocol described by the manufacturer. Rb1-bound HA concentration was calculated.

### Oligosaccharide binding assay

Oligosaccharide binding assay was performed as described previously [12]. Briefly, 50μl of 3μM biotinylated alpha N-acetyl neuaminosyl-2-3’-beta D-sialylgalactose (NeuAc-α2–3’-D-gal) was coated to a streptavidin-coated high-binding affinity microplate. 16 HAU of 2009pdm H1N1 was mixed with 500 and 1000 μg of Rb1 or HBSS (mock control). The mixture was then added to an oligosaccharide coated plate. Using anti-HA antibody and HRP-conjugated goat anti-mouse IgG secondary antibody, we quantitated the amount of virus attached to the oligosaccharide-coated plate.

### Statistical analyses

Data were analyzed by GraphPad Prism 6 software. Student’s-t test and one-way ANOVA were applied when comparing two different groups and more than two groups respectively. Survival differences were measured by log-rank test. P value less than 0.05 was considered significant.

## Results

### Antiviral activity of ginseng extracts (GE)

**Fig 1:**
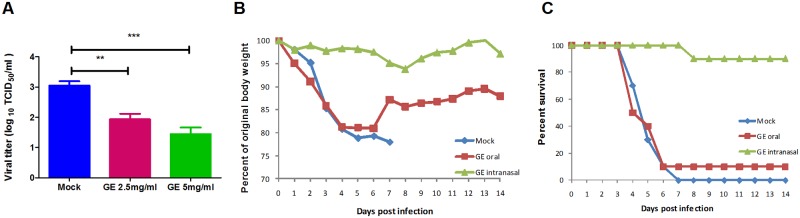
Antiviral activity of ginseng extract (GE) against 2009 pandemic H1N1 influenza A virus infection. (A) A dose dependent reduction in viral titer was observed when MDCK cells were infected with GE-pretreated A/Nanchang/8002/2009 (H1N1) virus. Protective effect of GE was observed on (B) weight loss and (C) survival of mice infected with A/Nanchang/8002/2009 (H1N1) virus. GE oral_ mice treated with 80mg/kg of GE by oral route. GE_ mice were infected with a mixture of 10^3^ EID_50_ of virus and GE. Mock_ there was no GE treatment given. *** P < 0.0005, ** P < 0.005.

In the virus-pretreatment assay, cells infected with GE-pretreated 2009pdm H1N1 virus showed a dose-dependent reduction of viral progeny in 24 hours of incubation (Fig 1A). No change in viral titers was observed when cells were treated with GE after infection (data not shown). The results indicate that GE might affect the viral life cycle at the beginning of infection. However we did not find any decrease in viral titer in cell-pretreatment assay, when cells were treated with GE prior to viral infection indicating that interaction of virus with the components presents in GE is necessary to obtain antiviral activity and immunostimulation of host cells might not be a reason behind the antiviral activity of GE.

Based on *in vitro* assays, we compared two different modes of GE treatment in BALB/c mice. The first group of animals was infected with 10^3^ EID_50_ of 2009pdm H1N1 (NC2) virus, which was pre-incubated with GE or vehicle for 1 hour at 37°C, while the second group was orally administrated with 80mg/kg of GE from day 0 to 7 post infection and infected with 10^3^ EID_50_ of NC2 virus. Mock controls received same amount of virus as GE treatment groups but no GE treatment was given. Interestingly, mice infected with the GE-NC2 mixture exhibited minimal loss in body weight with a 90% survival rate, which is significantly different from the results observed in the untreated and orally administered GE groups, which exhibited poor survival and more than 20% loss of body weight (P < 0.005) (Fig 1B and 1C). These results indicate that GE exhibits significant antiviral activity against 2009pdm H1N1 virus only if administered together with virus infection.

### Ginsenosides protect mice from lethal influenza virus infection

**Fig 2:**
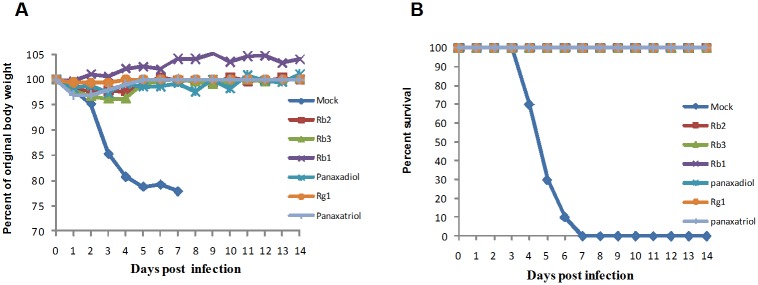
Protective effect of ginsenosides on lethal infection of 2009 pandemic H1N1 influenza virus infection in mice. Balb/c mice (n = 10/group) were infected with a mixture of A/Nanchang/8002/2009 (H1N1) and ginsenoside compounds such as Rb1, Rb2, Rb3, panaxadiol, Rg1 and panaxatriol or HBSS (mock) which was pre-incubated for 1 hour. Protective effect of ginsenosides was observed on (A) weight loss and (B) mortality of infected animals.

Since ginsenosides are bioactive compounds constituting more than 20% of Ginseng, we tested 8 different types of ginsenosides for antiviral activity in Balb/c mice. The treatment was given in a similar manner to that mentioned earlier for GE; briefly, 10^3^ EID_50_ of 2009pdm H1N1 virus was pre-incubated with ginsenosides Rb1, Rb2, Rb3, PPD, PPT, Rg1 or HBSS (mock control) for 1 hour and used to infect Balb/c mice. Animals were monitored daily for morbidity and mortality. Notably, the mice that received the ginsenoside-pretreated virus had minimal weight loss and no mortality, which was significantly different from the results seen in the mock control group, which showed severe weight loss and 100% mortality within 5 days post infection (P < 0.001) (Fig 2).

**Fig 3:**
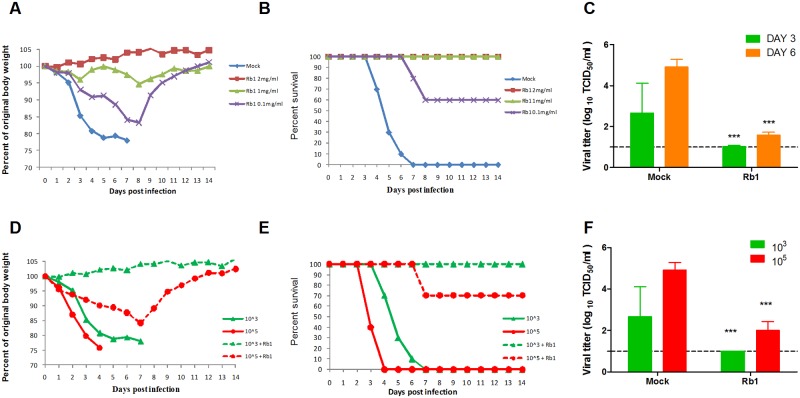
Dose dependent antiviral activity of ginsenoside Rb1. Balb/c mice (n = 10/group) were infected with 10^3^EID_50_ of A/Nanchang/8002/2009 (H1N1) pre-incubated with varying concentration of Rb1. Significant changes in (A) animal weight and (B) survival rate was observed in these group compared to mock (untreated) group. (C) MDCK cells were used to titrate viral loads present in lung tissues of Rb1(2mg/ml) treated and untreated animals at 3 and 6 dpi. (D-E) Mice (n = 10/group) were infected with different viral inoculum pre-incubated with 2mg/ml of Rb1. (D) changes in animal weight and (E) survival was observed in these group. (F) lung viral loads were titrated on 3 dpi (n = 3/group). Results were expressed as log10 values of the mean of viral load ± SEM. *** P < 0.0005.

Dose-dependent antiviral activity was assessed using Rb1 as a representative ginsenoside compound. In this experiment, we pre-incubated 10^3^ EID_50_ of 2009pdm H1N1 virus with different concentrations of Rb1 prior to animal infection. 2 mg and 1 mg/ml of Rb1 resulted in minimal weight loss and complete protection over lethal infection while 60% protection was found at 0.1mg/ml with a shift in the median day of death from 5 to 14 days post infection (P <0.001) (Fig 3A and 3B). Compared to untreated group, the mice lung virus titer was significantly decreased in the Rb1 treatment group by day 6 post infection. (Fig 3C). Thereafter, we compared the effectiveness of Rb1 on higher viral inoculum and increased the infectious dose of virus by 10^5^ EID_50_ for each animal. Data revealed moderate but significant weight loss and 70% animal survival in those animals that were infected with 10^5^ EID_50_ of the 2009pdm H1N1 and Rb1 mixture (Fig 3D and 3E). Significant reduction in lung virus titers of the Rb1 group was observed on day 3 post infection when compared with the titers in the mock control group (P < 0.001) (Fig 3F). Taken together, these data show that ginsenosides protect mice from lethal infection of 2009pdm H1N1 virus.

The activity of Rb1 was evaluated against different viruses such as PR8 and HK68 to characterize the range of antiviral activity. Eighty percent animals infected with PR8 survived in the Rb1 group compared to 30% in the vehicle group, with significant difference in weight loss (S1 Fig). Minimal weight loss was also observed in HK68 infected animals after Rb1 treatment (data not shown). The data showed that ginsenosides are active antiviral compounds present in ginseng.

### Interaction of Rb1 with virus is necessary for antiviral effect

Intranasal administration of ginsenosides might induce a localized immune response to combat viral particles. To test this hypothesis, we administered Rb1 and 2009pdm H1N1 virus to animals without prior incubation. The results showed that intranasal administration of Rb1 did not protect animals against lethal influenza infection. In addition, no significant difference in lung virus titers of Rb1 treated mice and those of the mock controls were observed (S2 Fig). The data confirm that only intranasal administration of Rb1 is not sufficient to protect the animals, whereas prior incubation of viral particles with Rb1 is critical for antiviral activity. Thereafter we performed a time dependent experiment, in which 2009pdm H1N1 was incubated with Rb1 for different time periods. We observed that Rb1, if incubated with virus for less than one hour, did not protect animals against lethal influenza infection (data not shown). The results imply that one-hour incubation might provide sufficient time for Rb1 to interact with viral particles and subsequently lead to interference in the viral life cycle.

### Rb1 interacts with viral hemagglutinin protein

**Fig 4:**
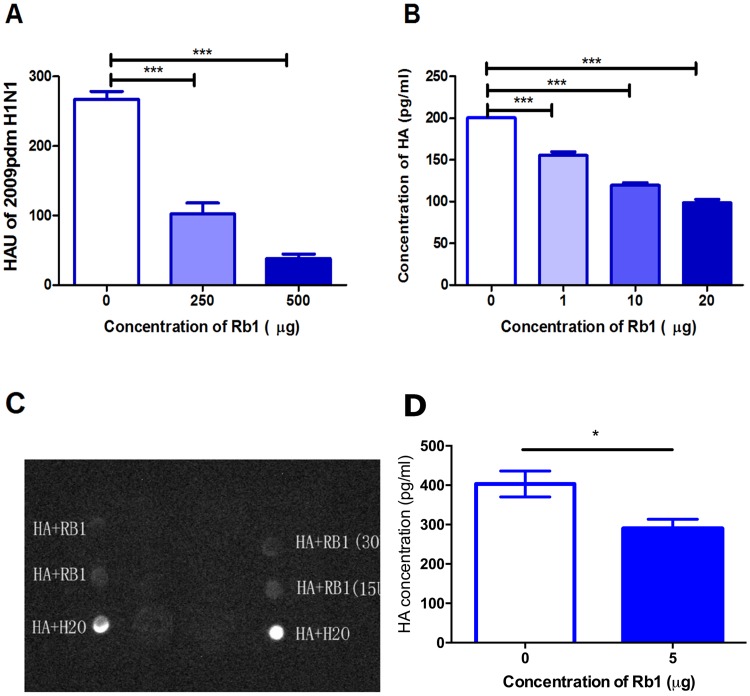
Interaction of Rb1 with viral hemagglutinin. (A) Serially diluted A/Nanchang/8002/2009 (H1N1) virus was pre-incubated with different concentrations of Rb1 or HBSS for 1 hour and used for hemagglutination inhibition assay. A significant reduction in HA titer of Rb1 treated virus was observed in dose dependent manner. To see the interaction of Rb1 with recombinant HA protein, HA was pre-incubated with different concentrations of Rb1 or H2O and assessed by (B) captured ELISA and (C) immunoblot assay. (D) Recombinant HA of H7N9 influenza virus was pre-incubated with 5μg of Rb1 and assessed by captured ELISA. Rb1 decreases the availability of HA protein for antibodies for anti-HA antibodies in dose dependent manner in panel B and C.

To determine which surface glycoprotein of influenza virus interacts with ginsenosides, hemagglutination inhibition (HAI) and neuraminidase inhibition (NAI) assays were performed with 2009 pdm H1N1 virus pre-incubated with different concentrations of Rb1 or HBSS (mock). A significant reduction in HA titer was observed in the presence of Rb1 in a dose-dependent manner (Fig 4A); however, no difference in enzymatic activity of neuraminidase was observed after Rb1 treatment (data not shown). The results indicate the possible interaction of ginsenosides with viral hemagglutinin. Fig 4B shows that recombinant HA-H1N1 protein bound with Rb1 limits the detection of HA protein by anti-HA-H1 antibodies in ELISA (P < 0.05). The quantitative analysis of Rb1 interaction with HA further shows that pre-incubation of recombinant HA protein with Rb1 reduces its availability for anti-HA-H1 antibody in a dose-dependent manner (Fig 4C). We further examined the binding Rb1 with HA-H7N9 virus. H7N9 is avian origin influenza A virus which is known for its binding specificity with α2–3 sialic acid receptor[13, 14]. As shown in Fig 4D, Rb1 interaction reduces the availability of H7-HA for HA specific antibody. The data confirm that Rb1 interacts with viral hemagglutinin proteins of different binding specificities. We could hypothesize that this interaction might interfere in the attachment of viral particles to the host cell surface.

### Rb1 prevent viral attachment with 2–3 sialic acid receptors

**Fig 5:**
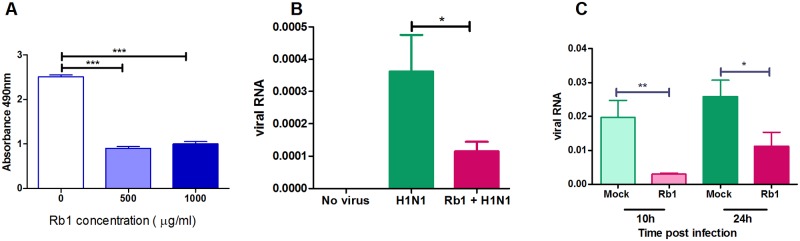
Rb1 prevents the attachment of virus with α2–3 sialic acid receptors on host cell surface. The attachment of Rb1 treated and untreated A/Nanchang/8002/2009 (H1N1) virus to α2–3’ sialic acid receptors was performed (A) by using alpha N-acetyl neuaminosyl-2-3’-beta D-sialylgalactose in oligosaccharide binding assay (B) by infecting CHO cells that predominantly contain α2–3’ sialic acid receptors at 4°C. The virus showed significantly less binding affinity in the presence of 500μg of Rb1 compared to mock control. (C) In replication assay, CHO cells were infected with Rb1 treated or untreated A/Nanchang/8002/2009 (H1N1) virus 37°C for 10 and 24 hours and subjected to real time RT-PCR. Rb1 treatment significantly inhibited 2009pdm H1N1 infection. *** P < 0.0005, ** P < 0.005, *P < 0.05.

Since HA is a key element for viral attachment to the host cell surface, we performed an oligosaccharide binding assay to evaluate the binding of 2009pdm H1N1 influenza virus to soluble sialic acid receptors in the presence of Rb1. The 2009pdm H1N1 influenza virus showed considerably less binding affinity to alpha N-acetyl neuaminosyl-2-3’-beta D-sialylgalactose (NeuAc- α 2–3’ -D-gal) residues in the presence of 500μg of Rb1 (P < 0.05) compared to mock control (Fig 5A). The NeuAc-α2–3’-D-gal serves as receptor preferentially binding with highly pathogenic avian influenza viruses. The data clearly suggest that ginsenosides inhibit viral attachment to α 2–3’ sialic acid receptors on the host cell surface.

We next quantitatively assessed viral particles attached to the host cell surface. CHO cells that predominantly contain α2–3’ sialic acid receptors were infected with Rb1-pretreated 2009pdm H1N1 virus and incubated at 4°C to prevent virus entry into the cell but still allow viral attachment to cellular receptors. Data analysis showed significantly decreased viral expression in the Rb1 group compared to viral expression in the mock controls (P < 0.05) (Fig 5B). Positive correlation of viral attachment was further observed on viral replication in CHO cells as Rb1 treatment significantly inhibited 2009pdm H1N1 infection 10 and 24 hours post infection (P < 0.05) (Fig 5C). Although 2009pdm H1N1 virus give binding preference to α2–6’ sialic acid receptors, it is already observed that being originated from swine and multiple influenza lineages, the virus has capability to bind with α2–3’ sialic acid receptors. We can hypothesize that Rb1 might lead to the inhibition of 2009pdm viral infection in α 2–3’ sialic acid containing cells.

### Sugar motifs are indispensible for antiviral activity of ginsenosides

**Fig 6:**
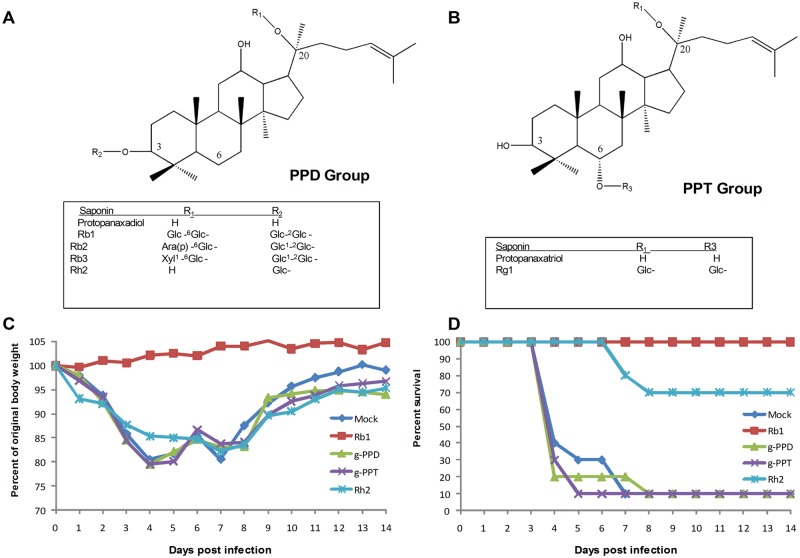
Sugar motifs are indispensible for antiviral activity of ginsenosides. The chemical structure (A) protopanaxadiol (PPD) and protopanaxatriol (PPT) show positions of sugar motifs present in their related saponins. Comparison of antiviral activity of Rb1 with carbohydrate-free metabolites of ginsenosides such as g-PPD and g-PPT was observed by (C) change in animal body weight and (D) mortality. Loss of antiviral activity was found with loss of sugar motifs.

Previous studies have demonstrated that upon ingestion, ginsenoside compounds undergo acid hydrolysis and a series of deglycosylation that result in the production of carbohydrate-free end metabolites such as g-PPD and g-PPT [7] [15]. To explore whether metabolic end products are involved in antiviral activity in vivo, we infected animals with 2009pdm H1N1 virus incubated with 2mg/ml of g-PPD, which is the end metabolite of Rb1, Rb2, Rb3 and g-PPT, the end metabolite of ginsenoside Rg1 (Fig 6A and 6B). Results showed no difference in animal morbidity and mortality between g-PPD, g-PPT and the control group (P > 0.05) (Fig 6C and 6D). We then tested ginsenoside Rh2 that has only carbohydrate moiety at position R1 and compared it with Rb1 that contain carbohydrate chains at two different positions (Fig 6A). Compared to Rb1, the group treated with Rh2 exhibited significant weight loss (P <0.05). 70% animals survived in Rh2 group, which is significantly different from mock control, g-PPD and g-PPT groups (P < 0.005) (Fig 6C and 6D). The data suggest that in vivo antiviral activity is actually exhibited by ginsenosides, not by their end-metabolites, and sugar motifs present in ginsenosides are indispensible for their antiviral nature.

## Discussion

In this study, we demonstrate that ginsenosides exert strong antiviral activity to 2009 pandemic H1N1 virus in vitro and in vivo. In vitro experiments describe that ginsenoside compounds, especially Rb1, has the ability to interact with viral hemagglutinin proteins, preventing the attachment of virus with α 2–3’ sialic acid receptors, subsequently leading to minimal viral entry into these cells and the decrease of viral infection. Furthermore, sugar motifs attached with a ginsenoside backbone are necessary for this interaction.

Interaction of an antiviral drug in the viral attachment process can be explained by two mechanisms: either the drug is able to interact directly with N-acetylneuraminic acid present on the cell surface, or bind to the surface proteins present on viral particles [16]. In this study, in vitro experiments showed that the addition ginsenoside Rb1 to MDCK cells prior to viral infection does not affect viral infection. In vivo experiments further showed that prior 1 hour incubation of the virus with Rb1 is prerequisite to seeing antiviral activity. We hypothesize that incubation of influenza virus might induce viral aggregation that tend to make conformational changes in viral surface proteins particularly in HA and reduce their infectivity to mammalian cells. In previous studies, preincubation of viral particles is observed to enhance antiviral and hemagglutinination inhibition activities of sialylated inhibitors[17–19]. Clearly, it is confirmed that Rb1 and other ginsenosides do not interact with host surface receptors to block viral entry.

This study shows physical interaction between Rb1 and this complex glycoprotein as possible mechanism for antiviral activity. The nature and stability of their interaction are not yet defined; however, we could hypothesize that binding between Rb1 and HA either develops conformational changes in HA proteins so they lose their affinity to sialic acid receptors, or Rb1 binds close to the binding pocket of the HA1 subunit, which leads to inhibition of the receptor binding process. To obtain the optimal antiviral activity of Rb1 or other ginsenosides, it is important to perform mechanistic studies in the future to develop a structure-based ginsenoside template with improved binding affinity and specificity to various influenza subtypes.

It is interesting that influenza-induced pathological changes in animal lungs existed during the acute course of infection, indicating the capability of virus to establish disease in the presence of Rb1; however, the magnitude of infection was lower. We further showed that Rb1 reduce influenza virus replication by inhibiting viral attachment to α 2–3’ sialic acid receptors. Given that 2009pdm H1N1 has the capability to bind with both α 2–3’ and α 2–6’ sialic acid receptors [20], while H3 and H1 viruses preferentially bind with α 2–6’ sialic acid receptors, we could speculate why Rb1 was more efficient in binding with 2009 pandemic H1N1. Although not complete but a significant decrease in H7N9-HA protein availability after Rb1 incubation supports our hypothesis. Specialized experiments with receptor binding sites of HA proteins should be performed to identify Rb1 interaction. The major limitation of this study is that we were unable to perform binding assays with Rb1 because of the unavailability of soluble α 2–6’ sialic acid. It is worthwhile to perform in vivo experiments to test Rb1 and other ginsenosides against avian influenza that cause severe disease in humans such as H5N1, H7N9 and H10N8.

In this study we demonstrate that magnitude of antiviral activity is related to the number of sugar motifs attached. Previous studies have shown that the anticancer activity of ginsenosides is inversely proportional to the number of sugar moieties [21]. Furthermore, the antioxidant activity of ginsenosides is related to the type and position of sugar moieties and the total number of hydroxyl groups attached [22]. These studies clearly suggest the strong role of sugar motifs in the biological properties of ginsenosides.

Ginseng is used worldwide as an alternative treatment of cough, cold and ILI without any side effects reported [23]. The pilot experiment performed in our laboratory did not result in negative or toxic effects of ginsenosides either in animals or in cell proliferation in vitro, thus defining the non-toxic nature and therapeutic value of these compounds on the concentrations tested. In phase II randomized clinical trials performed on children, oral consumption of ginseng extract as alternative influenza treatment did not result in severe adverse effects on human health [24]. It is anticipated that this study will lead to the development of a novel antiviral drug that might be useful to treat severe influenza infections.

## Supporting information

S1 Fig Ginsenosides Rb1 improved the survival of mice infected with A/Porto Rico/08/1934 (H1N1). Balb/c mice (n = 10/group) were infected with a mixture of A/Porto Rico08/1934 (H1N1) and Rb1 or HBSS (mock) which was pre-incubated for 1 hour. Protective effect of ginsenosides was observed on (A) weight loss and (B) mortality of infected animals.
(TIF)

S2 Fig Intranasal administration of Rb1 does not mice from lethal 2009 pdm H1N1. Balb/c mice (n = 10/group) were infected with 10^3^EID_50_ of A/Nanchang/8002/2009 (H1N1) and treated with intranasal administration of 2mg/kg of Rb1. No prior incubation of viral particles with Rb1 was given. No significant changes in (A) animal weight and (B) survival rate was observed in these group compared to mock (untreated) group. (C) MDCK cells were used to titrate viral loads present in lung tissues of Rb1 treated and untreated animals at 3 dpi.
(TIF)
