# Supplementary figures and images for: Inhibition of influenza A virus infection by ginsenosides
Source: PLoS One. 2017 Feb 10;12(2):e0171936. doi: 10.1371/journal.pone.0171936 (PMC5302443; doi:10.1371/journal.pone.0171936)

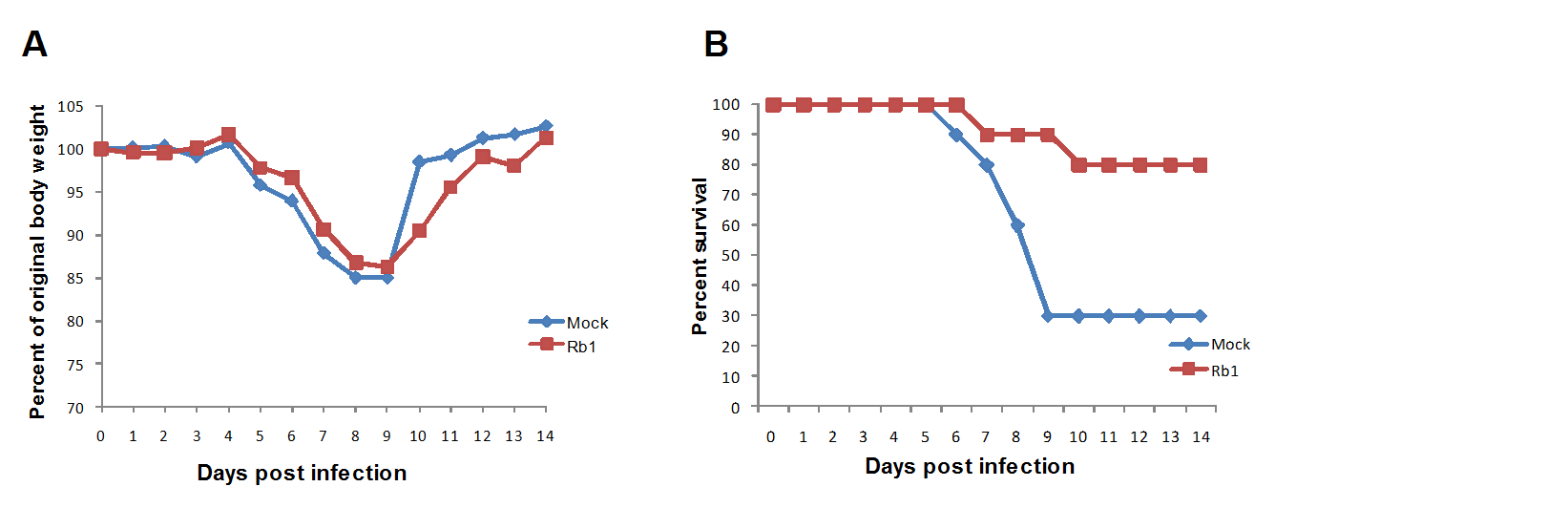

Supplement: S1 Fig — Balb/c mice (n = 10/group) were infected with a mixture of A/Porto Rico08/1934 (H1N1) and Rb1 or HBSS (mock) which was pre-incubated for 1 hour. Protective effect of ginsenosides was observed on (A) weight loss and (B) mortality of infected animals. (TIF) [file pone.0171936.s001.tif]

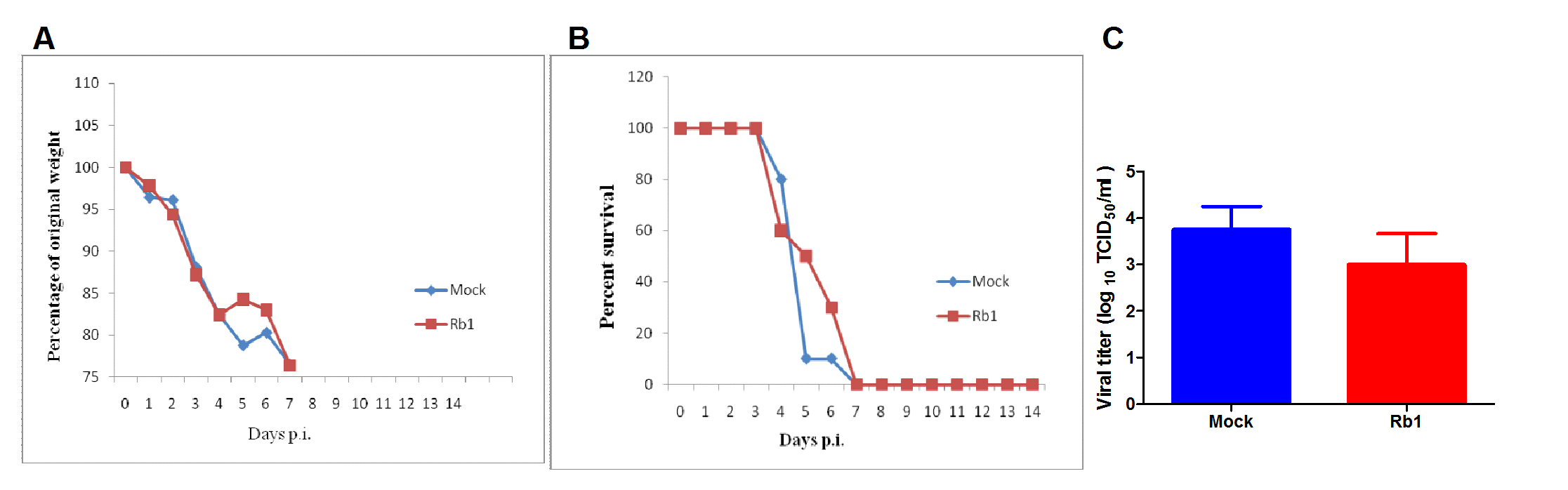

Supplement: S2 Fig — Balb/c mice (n = 10/group) were infected with 103EID50 of A/Nanchang/8002/2009 (H1N1) and treated with intranasal administration of 2mg/kg of Rb1. No prior incubation of viral particles with Rb1 was given. No significant changes in (A) animal weight and (B) survival rate was observed in these group compared to mock (untreated) group. (C) MDCK cells were used to titrate viral loads present in lung tissues of Rb1 treated and untreated animals at 3 dpi. (TIF) [file pone.0171936.s002.tif]
